# Health-related quality of life of advanced prostate cancer patients and spouses: results from actor-partner interdependence models

**DOI:** 10.1007/s00520-022-07100-8

**Published:** 2022-05-13

**Authors:** Christina Sauer, Andreas Ihrig, Tobias Hanslmeier, Johannes Huber, Kiriaki Hiller, Hans-Christoph Friederich, Imad Maatouk

**Affiliations:** 1 Department of General Internal and Psychosomatic Medicine, University Hospital Heidelberg, Im Neuenheimer Feld 410, 69120 Heidelberg, Germany; 2 National Center for Tumor Diseases (NCT), University Hospital Heidelberg, Heidelberg, Germany; 3 Department of Urology, Philipps University of Marburg, Marburg, Germany; 4 Department of Medical Oncology, National Center for Tumor Diseases (NCT), Heidelberg University Hospital, Im Neuenheimer Feld 460, 69120 Heidelberg, Germany; 5 Section of Psychosomatic Medicine, Psychotherapy and Psychooncology, Department of Internal Medicine II, Julius-Maximilian University Würzburg, Oberdürrbacher Straße 6, 97080 Würzburg, Germany

**Keywords:** Prostate cancer, Health-related quality of life, Psychological burden, Anxiety, Psychological interdependencies, Actor-partner interdependence model

## Abstract

**Background:**

Patients with prostate cancer (PC) and their spouses are confronted with several treatment-related and psychosocial challenges that can reduce their health-related quality of life (HRQoL). Patients with advanced PC (aPC) and their spouses are at highest risk for psychological distress and show lower HRQoL compared with couples in other phases. The aim of this study was to investigate the psychological interdependencies between HRQoL and anxiety, fear of progression (FoP), and depression in patients with aPC and their spouses.

**Methods:**

Ninety-six heterosexual couples with aPC participated in this cross-sectional study. Patients and spouses provided information about anxiety and depression (Patient Health Questionnaire-4), fear of progression (short form of the Fear of Progression Questionnaire), and HRQoL (EORTC QoL-C30, version 3). Psychological interdependencies were analyzed with various actor-partner interdependence models using structural equation modeling.

**Results:**

Anxiety, FoP, and depression were significant predictors of HRQoL for patients with aPC and their spouses (actor effects). Spouses’ anxiety and FoP were negatively associated with patients’ HRQoL (partner effects), showing that patients’ HRQoL is associated with their own and their spouses’ anxiety and FoP. No partner effect was revealed between depression and HRQoL in the patients or spouses.

**Conclusions:**

The resulted partner effects between spouses and patients underline the importance of considering HRQoL in patients with aPC from a dyadic perspective. It is important that physicians explore patients’ and spouses’ needs and psychological burden to offer support and access to psycho-oncological services. Future studies are needed to investigate the effects of suitable interventions on spouses’ anxiety and FoP.

**Supplementary Information:**

The online version contains supplementary material available at 10.1007/s00520-022-07100-8.

## Purpose

Prostate cancer (PC) is the second most common malignancy among men worldwide [1]. In recent decades, the relative 5-year survival has increased up to 93% in Germany [2] and 98% in the USA [3], due to better diagnostic and therapeutic approaches. Patients with PC are confronted with adverse treatment effects, such as sexual dysfunction, poorer bowel function, and urinary incontinence [4], as well as psychosocial challenges, such as increased depression [5], suicidal ideation [6], cancer-related anxiety [5, 7], fear of progression (FoP) [7], and distress [8], which reduce their health-related quality of life (HRQoL) [4, 9]. HRQoL is an increasingly important consideration for decision-making processes regarding therapies and their potential adverse effects, given that patients with localized and advanced PC (aPC) have various therapy options at their disposal [10]. PC is often described as a “partners” disease’ [11], reflecting this cancer type’s psycho-socio-sexual dimension (e.g., changes in the sexual and intimate relationship, disruptions in the usual social and domestic relationship, and dysfunctional communication with the partner [12]). Prior studies have suggested that spouses are even more psychologically burdened than patients with PC [13] and show lower emotional HRQoL [4]. Spouses are relevantly concerned about FoP [14] and show significantly higher FoP over the course of the disease [15]. Higher psychological distress and FoP might be explained by a general gender effect, showing that women reporting higher psychological distress than men regardless of their role [16].

Spouses are an important source of social support [17] which affects the emotional state [18] and HRQoL [19, 20] of patients with cancer. It is therefore crucial to investigate the psychological interdependencies between patients with PC and their partners. To investigate these interdependencies, analyses using the actor-partner interdependence model (APIM), a widely employed statistical model of dyadic relationships [21], are an appropriate approach. The results of an APIM with cancer survivor dyads from the USA (21.5% PC) have shown that partners’ FoP was significantly associated with the mental and physical HRQoL of patients and partners, while the patients’ FoP showed no association with the partners’ HRQoL [22]. A similar pattern revealed by a longitudinal study with 48 Asian couples showing that spouses’ prostate-specific anxiety and self-reported health status contributed to the physical HRQoL (but not the mental HRQoL) of the patients with PC [9]. Another study investigated the association between depression and FoP in couples with PC and laryngeal cancer and showed that partners’ depression predicts patients’ and partners’ FoP. These results might indicate that spouses’ higher psychological burden leads to spouses being less resourceful and supportive [22], which leads to more emotional symptoms and poorer HRQoL for patients with PC.

Despite the rising prevalence of PC, little research has been performed regarding couples with this disease. However, pair-relevant topics appear (e.g., sexuality after local therapy) even in the early stages of the disease [23]. On closer inspection, the investigation of these interdependencies is crucial but still pending, especially for patients with PC and their spouses in the advanced illness phase, who are at the highest risk for psychological distress and show lower HRQoL compared to couples in other phases [4]. Clinically, the identification of interdependencies underlines the need of noting and exploring the partners’ psychological burden of patients with aPC; furthermore, it is pivotal for the development and improvement of psycho-oncological interventions for patients *and* spouses with aPC that reduce the psychological burden and improve the HRQoL. Since patients with PC utilize less than patients with other cancer entities psycho-oncological services [24], the identification of factors that are associated with reduced HRQoL might help to find and justify other approaches to improve the HRQoL.

To the best of our knowledge, no study has investigated the interdependencies between anxiety/FoP/depression and HRQoL in patients with aPC and their spouses. We therefore aimed to fill this gap. Against the background of the reported studies, we hypothesized significant interdependencies (partner effects) only between spouses’ anxiety/FoP/depression and patients’ HRQoL, but not the other way round.

## Materials and methods

### Study design and sample

This study was a noninterventional explorative investigation and part of another study regarding therapy decision-making and psychological burden described elsewhere [25]. From October 2017 to February 2019, we conducted a cross-sectional survey of patients with aPC and their spouses treated at the National Center for Tumor Diseases (NCT), Heidelberg, Germany. The sample consisted of 96 heterosexual German couples. A recruiter asked the patients to participate in our study when the patients attended with their spouses. As general inclusion criteria, we defined aPC as locally advanced (T3-4) or metastatic (N+ or M+) prostate cancer, age ≥ 18 years, German as the mother tongue or excellent knowledge of German, and a written declaration of consent from both partners. The patients and their spouses completed their questionnaires either immediately or at home and returned them in a postage-paid envelope. Separate surveys were prepared for the patients and for their spouses, and they were asked to complete the surveys separately.

All participants provided written informed consent. The study complied with the Declaration of Helsinki, and the Ethics Committee of the University Clinic Centre Heidelberg approved the protocol (S-511/2017). We registered the study in the German Clinical Trial Register (Reference: DRKS 00013045).

### Measures

#### Depression and anxiety

The patients and their spouses completed the Patient Health Questionnaire-4 (PHQ-4), an ultrabrief tool with excellent psychometric properties [26] consisting of a 2-item depression scale (PHQ-2) and a 2-item anxiety scale (GAD-2). Scores for each subscales range from 0 to 6. The authors suggested considering scores of 3–4 as “yellow flags” and scores ≥ 5 as “red flags” for depression or anxiety [27].

#### Fear of progression

We assessed FoP with the short form of the Fear of Progression Questionnaire, which covers 4 subscales (affective reactions, partnership/family, occupation, loss of autonomy) with 12 items and shows excellent psychometric characteristics [28]. The cutoff point for determining treatment needs is ≥ 34 [29].

#### HRQoL

We used the quality of life (QoL)/global health status scale of the European Organization for Research and Treatment of Cancer [30] to assess QoL, which consists of 2 items (“How would you describe your overall physical condition during the last week?” and “How would you describe your overall quality of life during the last week?”). Values ranged from 0 to 100, with higher values representing better HRQoL.

### Statistical analysis

We performed the descriptive statistics and structural equation modeling with RStudio (version 1.3.959). We used the following R packages: haven, tidyr, dplyr, Hmisc, psych, and lavaan. A two-sided *p* < .05 was considered as statistically significant.

Structural equation modeling was employed to estimate the APIM model, a widely used model of dyadic relationships that takes into account the interdependence of two-person relationships with appropriate statistical techniques [23]. We estimated the parameter *k* (partner to actor effect ratio) to obtain an interpretable quantitative index that describes the relationship between the partner and actor effect (*k* = 1 suggests a couple pattern, *k* = 0 suggests an actor-only-pattern, and *k* = −1 suggests a contrast pattern) [31]^1^.

Given that the couples consisted of 2 distinguishable parts (i.e., women and men), we treated them as distinguishable. As recommended by Kenny and Ledermann [31], we analyzed the APIM with the following steps: in model 1, we estimated a saturated (just-identified) model; in model 2, we tested the equality of actor and partner effects; in model 3, we calculated the parameter *k* (partner to actor effect ratio) and bootstrap confidence intervals (CIs). For sensitivity analysis, we added the covariate age in the third models (see supplement 1). Since androgen deprivation therapy (ADT) is associated with higher risk of depression [32], we used *t*-tests for independent samples to investigate differences between patients with and without ADT (see supplementary file 2), and — in case of significant differences — controlled our models for ADT. For further sensitivity analyses, we added castration-resistance PC (CRPC) status (hormone-sensitive PC (HSPC) versus CRPC) to the models. The results of the sensitivity analyses are depicted in supplementary file 1.

Given that the QoL variables were not normally distributed (the results of the Shapiro-Wilk normality test were significant, *p*’s < .02), we used the maximum likelihood estimation with robust (Huber-White) standard errors and a scaled test statistic that is (asymptotically) equal to the Yuan-Bentler test statistic (estimator = “MLR”) to estimate models 1–3. Missing values were imputed by the full information maximum likelihood (“fiml”). As fit measures, we used the chi-squared test, the root mean square error of approximation (RMSEA, values ≤ .05 can be considered as a good fit), the standardized root mean square residual (SRMR, values ≤ .05 can be considered as a good fit, values ≤ .10 can be considered as an acceptable fit), and the comparative fit index (values > .97 can be considered as a good fit) [33]. Values are depicted in supplement 3.

## Results

### Patient and partner characteristics

Tables 1 and 2 list the participants’ characteristics and medical data. All participants were diagnosed with aPC, and the mean time from diagnosis was approximately 5 years. Eleven (11%) patients had no metastases and a T3 or T4 stage; 1 (1%) patient had only lymphogenic spread. In 8 (8.5%) patients, there was a history of secondary clinically relevant malignancy (except basal cell carcinoma) other than prostate cancer. A castration-sensitive tumor was diagnosed in 55 (57%) patients. CRPC or using ADT was associated with significant higher depression and lower HRQoL (see supplement 2). We also investigated differences between patients with simple ADT (*n* = 48) and maximal androgen blockade^2^ (MAB, *n* = 32) and did not find any differences in anxiety, FoP, or depression levels (all *p*’s > .46. results are not depicted). All spouses were female. Couples were in a permanent partnership for a mean of 37.5 years (*SD*, 13.5; range, 2–58 years); 94 (98%) couples lived together; 88 (92%) were married; and 81 (84%) had children. The spouses were significantly younger than the patients. No relevant differences were revealed in education level. In terms of work status, the patients were more often retired, and the spouses were more often still employed. One spouse reported the regular use of antidepressant medication (and *n* = 1 the use on demand), and one spouse reported the regular use of medication against anxiety (and *n* = 1 on demand). Regarding the psychosocial symptoms, the spouses showed significantly higher anxiety and FoP levels than the patients; no differences were revealed between depression and HRQoL scores. Spouses of patients using ADT showed significant higher FoP and lower HRQoL than spouses of patients using no ADT (see supplementary file 2).

**Table 1:** Descriptive statistics of the sample

	Patient *n* = 96	Spouse *n* = 96	
	***n (%)***	***n (%)***	*χ*^*2*^ (2)
Education			5.55
Elementary school	43 (44.8)	40 (41.7)	
Middle school	15 (15.6)	28 (29.2)	
High school	38 (29.6)	28 (29.2)	
Work status			21.04***
Employee	19 (19.8)	43 (45.3)	
Pension	64 (66.7)	51 (53.7)	
On sick leave	13 (13.5)	1 (1.1)	
	***M (SD)***	***M (SD)***	*t*
Age	69.05 (9.01)	64.46 (9.16)	3.47***
Psychosocial symptoms			
GAD-2	1.35 (1.40)	1.94 (1.59)	−2.70***
PHQ-2	1.79 (1.64)	1.84 (1.45)	−0.23
FoP	30.87 (8.85)	34.07 (9.86)	−2.36*
HRQoL	55.47 (22.58)	57.20 (19.48)	−0.57

GAD-2 (anxiety scale) and PHQ-2 (depression scale) of the Patient Health Questionnaire-4 (PHQ-4)

*M* mean, *SD* standard deviation, *FoP* fear of progression, *HRQoL* health-related quality of life

*p <.05; ***p ≤ .001

**Table 2:** Patients’ disease-related parameters (*n* = 96)

	Mean (*SD*), range
Months since diagnosis^1^	61 (60.1), 1–265
	*n* (%)
Metastasis	84 (87.5)
Affected lymph nodes	55 (57.3)
Gleason score >7	60 (62.5)
T3 or T4 staging	53 (55.2)
CRPC-status^1^	
CRPC	52 (54.7)
HSPC	43 (45.3)
Hormone therapy (yes/no)^1^	80 (84)
ADT	48 (50.5)
MAB	32 (33.7)
No ADT	15 (15.8)
Further treatments (ongoing or completed)^1^	
Chemotherapy	36 (38.9)
Radiotherapy	42 (44.2)
Prostatectomy	53 (55.8)
Psychopharmaca^1^	
No	85 (89.5)
Antidepressants	5 (5.3)
Pain medication	2 (2.1)
Others	3 (3.3)
Secondary diagnosis	
Psychiatric diagnosis^2^	
No	87 (95.5)
Depression	3 (3.3)
Anxiety disorder	2 (2.2)
Diabetes mellitus (DM)^1^	
No	79 (83.2)
Yes	16 (16.8)
Arterial hypertension^1^	
No	40 (42.1)
Yes	55 (57.9)
Cardiac disease^1^	
No	78 (82.1)
Yes	17 (17.9)
Dysrhythmia	
No	81 (85.3)
Yes	14 (14.7)
Cerebrovascular disease^1^	
No	90 (94.7)
Vascular	4 (4.2)
Cerebral	1 (1.0)
Malignoma^1,3^	
No	87 (91.6)
1	5 (5.3)
2	3 (3.2)

*SD* standard deviation, *CRPC* castration-resistant prostate cancer, *HSPC* hormone-sensitive prostate cancer, *ADT* androgen deprivation therapy, *MAB* maximal androgen blockade

^1^One missing value

^2^Four missing values

^3^Clinically relevant malignancy (except basal cell carcinoma) other than prostate cancer

### Correlation matrix

The correlation matrix (available in the supplementary file 4) shows that all correlations of psychosocial symptoms (GAD, FoP, PHQ, and HRQoL) in and between the patients and spouses were statistically significant.

### APIM

**Fig. 1:**
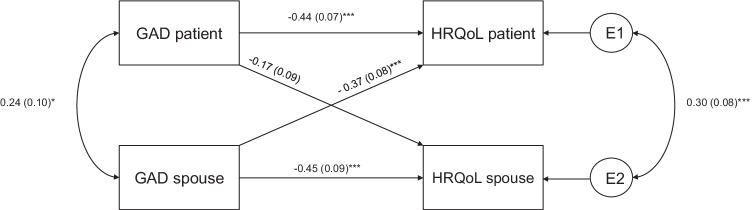
APIM (standardized estimations) for GAD on HRQoL. GAD, general anxiety disorder; HRQoL, health-related quality of life. *p < .05; ***p < .001

**Fig. 2:**
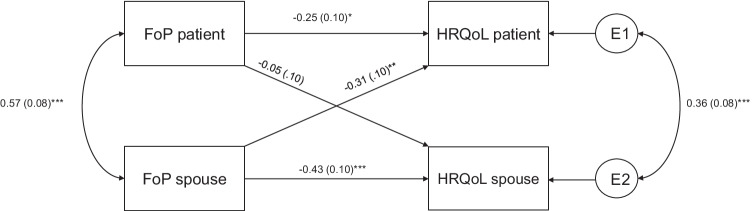
APIM (standardized estimations) for FoP on HRQoL. FoP, fear of progression; HRQoL, health-related quality of life. *p < .05; **p < .01; ***p < .001

**Fig. 3:**
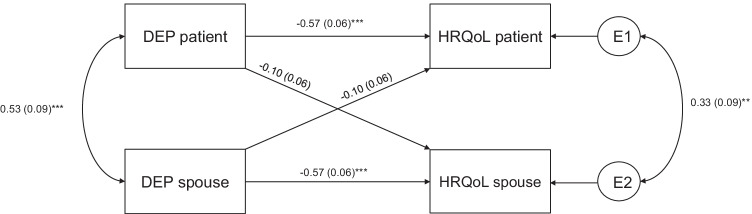
APIM (equal actor and partner effects) for depression on HRQoL. Dep, depression; HRQoL, health-related quality of life. **p < .01; ***p < .001

Three separate APIMs were conducted (GAD-QoL, FoP-QoL, and Depression-QoL) with standardized estimators. The results of the respective models are depicted in Figs. 1, 2, 3 (see supplement 3 for fit parameters). The results of the models with the covariates age, CRPC status, and ADT are provided in supplement 1. Sensitivity analyses revealed that patients’ CRPC status, but not ADT showed a significant effect on patients HRQoL. Neither adding CRPC status nor ADT changed the reported results. Adding the covariate age did not change the reported results^3^ but show poor parameter fit (e.g., comparative fit index < .67) (see supplement 1).

#### GAD

We found significant actor effects in the patients and their spouses and a significant partner effect for spouses’ GAD on the patients’ HRQoL (Fig. 1). The partner effect for patients’ GAD on the spouses’ HRQoL reached no significance (*p* = .069). The model explains 40% of the patients’ and 26% of the spouses’ variance in QoL. Calculating the *k* supported a couple pattern for the patients’ QoL (*k* = 0.83; *CI* = [0.27; 1.40]) and an actor-only pattern for the spouses’ QoL (*k* = 0.38; *CI* = [−0.10; 0.85]). The patients’ HRQoL was therefore associated with their own and their spouse’s GAD, whereas the spouses’ HRQoL was associated only with their own GAD.

#### FoP

The APIM revealed significant actor effects in the patients and their spouses and a significant partner effect for spouses’ FoP on the patients’ HRQoL (Fig. 2). The model explained 25% of the patients’ and 21% of the spouses’ variance in HRQoL. Calculating the *k* supported a couple pattern for the patients’ QoL (*k* = 1.23; *CI* = [−0.32; 2.78]) and an actor-only pattern for the spouses’ QoL (*k* = 0.11, *CI* = [−0.40; 0.61]). The patients’ HRQoL was therefore associated with their own and their spouse’s FoP, whereas the spouses’ HRQoL was associated only with their own FoP.

#### Depression

Given that model 2 revealed the equality of the actor and partner effects (*χ*^*2*^= 1.92, *df* = 2, *p* = .38; SABIC_model2_ < SABIC_model1_), we set the actor and partner effects as equal. We found a significant actor effect (*ß* = −0.56 [0.060], *p* = .000) but no partner effects (*ß* = −0.098 [0.060], *p* = .108) (see Fig. 3). The model explained 39% of patients’ and spouses’ variance in QoL. Calculating the *k* underlines the actor-only pattern (*k* = 0.173; *CI* = [−0.05; 0.40]).

## Discussion

The aim of this cross-sectional study was to examine the psychological interdependencies between anxiety, FoP, depression, and HRQoL of patients with aPC and that of their spouses. Our main finding showed that spouses’ anxiety and FoP were negatively associated with the patients’ HRQoL (partner effects), showing that the patients’ and spouses’ anxiety and FoP contributed to the patients’ HRQoL, whereas only the spouses’ anxiety and FoP contributed to their own HRQoL. There was no association between the spouses’ depression and the patients’ HRQoL (actor-only pattern). Our hypotheses were therefore partially confirmed.

### Effect of anxiety and FoP on HRQoL

The results of both APIMs revealed a remarkable partner effect for anxiety and FoP on the patients’ HRQoL, suggesting that the spouses’ anxiety and FoP were substantially associated with HRQoL of the patients with aPC. This result is in line with our hypothesis and the results of a previous study [9] suggesting that increased psychological burden in the spouses of patients with aPC might lead to less social support, which could lead to poorer HRQoL in patients with aPC. This hypothesis is supported by the results of a longitudinal study of patients with PC and their spouses that showed that spouses support and having the patients feeling supported significantly predicted the patients’ QoL and relationship satisfaction [20]. Moreover, previous research on patients with PC has shown that a lack of positive support and detrimental interactions were associated with poorer mental QoL [34]. A more accurate hypothesis for our results might be that the increased anxiety and FoP of spouses of patients with aPC (due to the heightened awareness of their partners’ life-threating illness and mortality) lead to more detrimental interactions that remind patients on their own life-threating illness and mortality, which could decrease their HRQoL. Given that we do not know how the spouses’ anxiety and FoP were expressed within the relationship, this hypothesis should be considered with caution. In a cross-sectional study, Regan et al. showed that the supportive dyadic coping mechanism of spouses (patients with PC and their spouses) was positively associated with anxiety and depression [35]. This might suggest that spouses’ increased anxiety elicits more supportive dyadic coping from patients with aPC, which could deplete personal resources and therefore deteriorate HRQoL. Further studies are needed to investigate the moderators and mediators (different aspects of social support, emotional expression, coping processes, relationship satisfaction) of the association between anxiety, FoP, and HRQoL in patients with aPC and their spouses.

### Effect of depression on HRQoL

In contrast to the APIMs with anxiety and FoP, the actor and partner effects in patients and spouses were equal. The lower depression scores of the patients and spouses were associated with better HRQoL (negative actor effect). Against our hypothesis, we found no partner effect of depression on HRQoL, which is in line with a study of Chinese patients with different cancer entities (but no PC) and family caregivers [36], which showed no significant partner effects of depression and anxiety on the physical and mental component score of the Short-Form Survey (SF-12). However, on closer inspection of the eight SF-12 subscales, caregiver anxiety predicted the patients’ general health, a finding that might reveal the importance of differentially investigating the effects of anxiety and depression on the various components of HRQoL so that exact conclusions can be drawn.

Nonpathological and comparable PHQ-2 scores might be one reason for the lack of partner effects on depression in HRQoL. When compared with the results of Regan et al. [35], our findings might suggest that congruent nonclinical emotional states do not require increased supportive dyadic coping (see explanation above). Future studies of patients with aPC and their spouses are needed to investigate the dyadic dynamics of depression in the various components of HRQoL.

### Clinical implications

Our findings show that the anxiety and FoP of spouses of patients with aPC are associated with the patients’ and their own HRQoL. The findings underline the importance of psycho-oncological interventions that reduce spouse anxiety and FoP. Previous research has shown the caregivers’ needs for psycho-oncological support for reducing FoP [37]. It is important that physicians consider and explore the spouses’ needs and psychological burden during the consultation and, if necessary, offer support and referral to psycho-oncological services.

Psycho-oncological interventions reveal small to medium effects in reducing FoP in patients with cancer [38]. However, a systematic review [39] revealed that only 1 psycho-oncological intervention showed a positive impact on anxiety (and depression) for spouses of patients with cancer. For spouses of men with PC in particular, another review [40] showed that of 11 existing interventions for spouses, 6 focused on relieving distress and improving emotional well-being, and only 1 focused on FoP, among other themes. However, no study has investigated the effects of anxiety or FoP, and only 1 study has shown improvements in depression. Interventions for spouses focusing on anxiety and FoP, especially for spouses of patients’ in the advanced stage, are therefore lacking. The results of a current review showed that contemporary cognitive behavioral therapies (e.g., acceptance and commitment therapy [41], mindfulness-based stress reduction [42]) show larger effects than classical cognitive behavioral approaches in reducing the FoP of patients with cancer [38], which might indicate that these therapeutic approaches are also effective for reducing FoP in spouses.

Results are still pending as to whether couple interventions or interventions for spouses alone are more effective for reducing psychological distress and improving HRQoL of aPC dyads [40]. Since we found actor and partner effects (i.e., patients HRQoL was associated with their own and their partners’ anxiety and FoP), and patients with PC show a lower utilization of psycho-oncological support than patients with other tumor entities [24], our results might be a further evidence for couple interventions, which could also target the couple dynamics and psychosocial burden of patients and might reduce barriers of the utilization of psycho-oncological support. In line with this, previous research speaks in favor of couple interventions. The findings from a study of patients with aPC and their spouses showed that dyadic coping processes increase resiliency in couples coping with psychological distress [43]. Another study showed that the problem-focused and emotion-focused coping and social support seeking of spouses of patients with PC longitudinally predicted the low mental HRQoL of patients with PC [44]. Additionally, a recent meta-analysis showed small to medium effects sizes of dyadic interventions for caregivers and patients (including lifestyle interventions like physical training) with regard of improvements in the emotional, social, spiritual, and mental aspects of patient quality of life, in their relationships with caregivers, marital functioning, depression, and anxiety [45]. In summary, our results underline the need for interventional studies that investigate dyadic interventions that reduce the anxiety and FoP of spouses of patients with aPC.

### Strength and limitations

Our study has several important strengths and extends the prior research on psychological interdependencies in the HRQoL of patients with PC and their spouses. The first major strength is the data frame with 96 patient/spouse dyads. The second is that this is, to the best of our knowledge, the first study to investigate the psychological interdependencies between anxiety/FoP/depression and HRQoL of patients with PC and their spouses in the advanced illness phase. The third strength lies in the statistical analyses with APIMs, which allows the precise estimation of regression parameters and analyze interdependencies. Nevertheless, we are also aware of our study’s limitations. Given that this was a cross-sectional study design, causality and directionality cannot be implied. We investigated 96 German patient/spouse dyads with aPC and, although it was not an inclusion criterion, only heterosexual couples participated in our study. The generalizability of our results to homosexual couples or couples in other illness phases or of other ethnicities might therefore be limited. Moreover, we assessed HRQoL as 1 global construct with only 2 items; however, no differentiation between mental QoL and physical QoL can be drawn. Future studies should investigate the association between psychological symptoms and mental versus physical QoL or PC-specific QoL (e.g., assessed with the European Organization for Research and Treatment of Cancer HRQoL Questionnaire Prostate Cancer module). Symptoms of anxiety and depression were assessed by the PHQ-4, a widely used and validated ultrabrief screening tool. However, future studies should insert longer instruments, e.g., the PHQ-9 and the GAD-7. Lastly, as explained above, future studies are needed to investigate the moderators and mediators (e.g., quality of the relationship, dyadic coping) between psychological symptoms and HRQoL in patients with PC and their spouses.

## Conclusion

The results of this study highlight the importance of considering HRQoL in patients with aPC from a dyadic perspective in clinical contexts. Psychologically interdependencies were shown between spouses’ anxiety and FoP and patients’ HRQoL, i.e., patients’ and spouses anxiety and FoP contributed to the patients’ HRQoL. Physicians should note and explore the psychological well-being of spouses during the consultation and, if indicated, offer couple-based psycho-oncological interventions that target anxiety and FoP of the spouses of patients with aPC. This might be a pivotal step for improving the HRQoL of patients with aPC and of their spouses. Acceptance- and mindfulness-based interventions and lifestyle interventions might be a promising approach for reducing anxiety and FoP of these spouses. Future randomized controlled intervention studies are needed to investigate the effects of these interventions on anxiety and FoP in the spouses and dyads of patients with aPC.

## Supplementary Information

ESM 1 (DOCX 28.1 kb)

ESM 2 (DOCX 16.6 kb)

ESM 3 (DOCX 15.7 kb)

ESM 4 (DOCX 16.5 kb)

## Data Availability

Data are available upon request by the first author.

## Footnotes

1 To estimate *k*, we also calculated the bootstrapped CIs (number of bootstraps = 5000), which did not change the reported results.

2 MAB included a combination of medical (GnRH-agonist, GnRH-antagonist) or surgical castration (such as subcapsular orchiectomy, ablatio testis) with a nonsteroidal antiandrogen (such as Bicalutamide, Flutamide, Nilutamide) or steroidal androgen (Cyproteronacetat) or new hormonal agents (such as Enzalutamide, Abiraterone).

3 One exception in GAD APIM: partner effect GAD_patient_ → HRQoL_spouse_ gets significant, *p* = .039. Since the model fit is very poor, CFI = .623, SRMR = .150, this result must be interpreted with caution.

## Code availability

Code is available upon request by the first author.
