# Supplementary material for: Health-related quality of life of advanced prostate cancer patients and spouses: results from actor-partner interdependence models
Source: Support Care Cancer. 2022 May 13;30(8):6985–93. doi: 10.1007/s00520-022-07100-8 (PMC9213378; doi:10.1007/s00520-022-07100-8)
Supplement: Supplementary file 1 — (DOCX 28.1 kb) [file 520_2022_7100_MOESM1_ESM.docx]

Supplement 1: Sensitivity Analyses:

APIMs controlled for

1. age
2. CRPC status (CRPC versus HSPC)
3. ADT (ADT versus no-ADT)
4. **Age**

Model General Anxiety Disorder (GAD)

|  | *β (SE)* | *p* |
| --- | --- | --- |
| actor-effect |  |  |
| GAD_patient_ | - .46 (.08) | < .001 |
| GAD_spouses_ | - .46 (.08) | < .001 |
|  |  |  |
| partner-effect |  |  |
| GAD_patient_ | - .37 (.08) | .039 |
| GAD_spouses_ | - .18 (.09) | < .001 |
|  |  |  |
| age ->HRQoL |  |  |
| age_patient_ | - .08 (.08) | .307 |
| age_spouses_ | - .13 (.08) | .125 |
|  |  |  |
| age ->HRQoL |  |  |
| patient | - .23(.12) | .098 |
| spouses | - .11(.10) | .312 |

CFI = .623, SRMR = .150

Model Fear of Progression (FoP)

|  | *β (SE)* | *p* |
| --- | --- | --- |
| actor-effect |  |  |
| FoP_patient_ | - .29 (.10) | .006 |
| FoP_spouses_ | - .41 (.10) | < .001 |
|  |  |  |
| partner-effect |  |  |
| FoP_patient_ | - .09 (.12) | . 006 |
| FoP_spouses_ | - .29 (.10) | . 462 |
|  |  |  |
| age ->HRQoL |  |  |
| age_patient_ | - .08 (.10) | .432 |
| age_spouses_ | - .12 (.10) | .234 |
|  |  |  |
| age -> FoP |  |  |
| patient | - .35 (.09) | .001 |
| spouses | - .04 (.09) | .661 |

CFI = .669, SRMR = .162

Model Depression (PHQ-2)

|  | β (SE) | p |
| --- | --- | --- |
| actor-effect |  |  |
| PHQ_patient_ | - .57 (.06) | < .001 |
| PHQ_spouses_ | - .57 (.06) | < .001 |
|  |  |  |
| partner-effect |  |  |
| PHQ_patient_ | - .10 (.06) | .101 |
| PHQ_spouses_ | - .10 (.06) | .101 |
|  |  |  |
| age ->HRQoL |  |  |
| age_patient_ | - .07 (.06) | .239 |
| age_spouses_ | - .06 (.06) | .336 |
|  |  |  |
| age -> PHQ-2 |  |  |
| patient | - .07 (.11) | .495 |
| spouses | .00 (.11) | .979 |

CFI = .621, SRMR = .165

1. **CRPC status**

Model GAD

|  | β (SE) | p |
| --- | --- | --- |
| actor-effect |  |  |
| GAD_patient_ | - .42 (.08) | < .001 |
| GAD_spouses_ | - .45 (.09) | < .001 |
|  |  |  |
| partner-effect |  |  |
| GAD_patient_ | - .17 (.09) | .069 |
| GAD_spouses_ | - .35 (.08) | <. 001 |
|  |  |  |
| covariate CRPC |  |  |
| patient | .24 (0.07) | .002 |

CFI = .996, SRMR = .057

Model FoP

|  | β (SE) | p |
| --- | --- | --- |
| actor-Effect |  |  |
| FoP_patient_ | - .29 (.10) | .002 |
| FoP_spouses_ | - .43(.10) | < .001 |
|  |  |  |
| partner-effect |  |  |
| FoP_patient_ | - .04 (.10) | .677 |
| FoP_spouses_ | - .25 (.11) | .023 |
|  |  |  |
| covariate CRPC |  |  |
| patient | .30 (.08) | <. 001 |

CFI = .997, SRMR = .042

Model Depression

|  | β (SE) | p |
| --- | --- | --- |
| actor-effect |  |  |
| PHQ_patient_ | - . 55 (.06) | < .001 |
| PHQ_spouses_ | - .56 (.06) | < .001 |
|  |  |  |
| partner-effect |  |  |
| PHQ_patient_ | - .11 (.06) | .075 |
| PHQ_spouses_ | - .11 (.06) | .075 |
|  |  |  |
| covariate CRPC |  |  |
| patient | - .11 (.06) | .082 |

CFI = .977 , SRMR = .083

1. **Androgendeprivation therapy (ADT)**

Model GAD

|  | *β (SE)* | *p* |
| --- | --- | --- |
| actor-effect |  |  |
| GAD_patient_ | - .43 (.07) | < .001 |
| GAD_spouses_ | -.45 (.09) | <. 001 |
|  |  |  |
| partner-effect |  |  |
| GAD_patient_ | -.17 (.09) | .069 |
| GAD_spouses_ | -.36 (.08) | < .001 |
|  |  |  |
| covariate ADT -> HRQoL |  |  |
| patient | - .10 (.06) | .098 |

CFI = .959, SRMR = .081

|  | *β (SE)* | *p* |
| --- | --- | --- |
| actor-effect |  |  |
| GAD_patient_ | - .44 (.07) | < .001 |
| GAD_spouses_ | -.44 (.09) | <. 001 |
|  |  |  |
| partner-effect |  |  |
| GAD_patient_ | -.16 (.09) | .085 |
| GAD_spouses_ | -.37 (.08) | < .001 |
|  |  |  |
| covariate ADT -> HRQoL |  |  |
| spouse | - .07 (.08) | .337 |

CFI = .938, SRMR = .085

Model FoP

|  | *β (SE)* | *p* |
| --- | --- | --- |
| actor-effect |  |  |
| FOP_patient_ | - .26 (.10) | .008 |
| FOP_spouses_ | - .43 (.10) | < .001 |
|  |  |  |
| partner-effect |  |  |
| FOP_patient_ | - .04 (.10) | .670 |
| FOP_spouses_ | - .29 (.10) | .008 |
|  |  |  |
| covariate ADT -> HRQoL |  |  |
| patient | - .11 (.06) | .068 |

CFI = .940, SRMR = .90

|  | *β (SE)* | *p* |
| --- | --- | --- |
| actor-effect |  |  |
| FOP_patient_ | - .25 (.10) | .010 |
| FOP_spouses_ | - .42 (.10) | < .001 |
|  |  |  |
| partner-effect |  |  |
| FOP_patient_ | - .05 (.11) | .661 |
| FOP_spouses_ | - .31 (.10) | .003 |
|  |  |  |
| covariate ADT -> HRQoL |  |  |
| spouse | - .04 (.08) | .577 |

CFI = .910, SRMR = .097

|  | *β (SE)* | *p* |
| --- | --- | --- |
| actor-effect |  |  |
| FOP_patient_ | - .25 (.10) | .009 |
| FOP_spouses_ | - .42 (.10) | < .001 |
|  |  |  |
| partner-effect |  |  |
| FOP_patient_ | - .04 (.10) | .671 |
| FOP_spouses_ | - .31 (.10) | .003 |
|  |  |  |
| covariate ADT -> FoP |  |  |
| spouse | .19 (.08) | .021 |

CFI = .963, SRMR = .066

Model Depression

|  | *β (SE)* | *p* |
| --- | --- | --- |
| actor-effect |  |  |
| PHQ_patient_ | - .56 (.06) | < .001 |
| PHQ_spouses_ | - .57 (.06) | < .001 |
|  |  |  |
| partner-effect |  |  |
| PHQ_patient_ | - .10 (.06) | .106 |
| PHQ_spouses_ | - .10 (.06) | .106 |
|  |  |  |
| covariate ADT ->HRQoL |  |  |
| patient | - .05 (.04) | .188 |
|  |  |  |
| covariate ADT -> PHQ |  |  |
| patient | .19 (.07) | .012 |

CFI = 1.00, SRMR = .094

|  | *β (SE)* | *p* |
| --- | --- | --- |
| actor-effect |  |  |
| PHQ_patient_ | - .57 (.06) | < .001 |
| PHQ_spouses_ | - .57 (.06) | < .001 |
|  |  |  |
| partner-effect |  |  |
| PHQ_patient_ | - .10 (.06) | .103 |
| PHQ_spouses_ | - .10 (.06) | .103 |
|  |  |  |
| covariate ADT ->HRQoL |  |  |
| spouse | . 02 (.04) | .678 |

CFI = .951, SRMR = .125
