# Supplementary material for: Health-related quality of life of advanced prostate cancer patients and spouses: results from actor-partner interdependence models
Source: Support Care Cancer. 2022 May 13;30(8):6985–93. doi: 10.1007/s00520-022-07100-8 (PMC9213378; doi:10.1007/s00520-022-07100-8)
Supplement: Supplementary file 2 — (DOCX 16.6 kb) [file 520_2022_7100_MOESM2_ESM.docx]

| **CRPC-status** | HSPC  *Mean (SD)*  *n* = 43 | CRPC  *Mean (SD)*  *n* = 52 | T/p |  |
| --- | --- | --- | --- | --- |
| **patients** |  |  |  |  |
| GAD-2 | 1.09 (1.31) | 1.52 (1.42) | 1.51/0.135 |  |
| PHQ-2 | 1.40 (1.42) | 2.04 (1.68) | **1.99/0.049** |  |
| FoP | 30.60 (9.10) | 30.75 (8.43) | 0.78/0.938 |  |
| HRQoL | 64.15 (18.50) | 49.36 (22.50) | **−3.45/0.001** |  |
|  |  |  |  |  |
| **spouses** |  |  |  |  |
| GAD-2 | 1.70 (1.46) | 2.06 (1.60) | 1.14/0.259 |  |
| PHQ-2 | 1.86 (1.47) | 1.75 (1.34) | −.38/.0703 |  |
| FoP^1^ | 32.26 (9.23) | 35.25 (10.02) | 1.50/0.138 |  |
| HRQoL | 58.91 (20.44) | 56.73 (17.70) | −.56/0.578 |  |
|  | | | | |
| **ADT (yes, no)** | yes  (*n* = 80) | no  (*n* = 15) |  |  |
| **patients** |  |  |  |  |
| GAD-2 | 1.41 (1.38) | 0.87 (1.30) | −1.41/0.161 |  |
| PHQ-2 | 1.91 (1.62) | 0.87 (1.13) | **−2.39/0.019** |  |
| FoP^1^ | 31.16 (8.96) | 28.13 (6.78) | −1.24/0.217 |  |
| HRQoL | 53.75 (22.30) | 68.33 (15.49) | **2.42/0.017** |  |
|  |  |  |  |  |
| **spouses** |  |  |  |  |
| GAD-2 | 2.00 (1.60) | 1.33 (1.05) | −1.55/0.125 |  |
| PHQ-2 | 1.85 (1.38) | 1.53 (1.51) | −.81/0.423 |  |
| FoP^1^ | 34.97 (9.65) | 28.13 (8.29) | **−2.57/0.012** |  |
| HRQoL | 56.04 (18.75) | 66.67 (17.82) | **2.03/0.045** |  |

Supplement 2: Results of t-Tests between patients/spouses under different medical conditions

^1^ one missing value. *SD*, standard deviation; CRPC = castration-resistant prostate cancer. HSPC = hormon-sensitive prostate cancer. ADT = androgen deprivation therapy.
