# Supplementary material for: Health-related quality of life of advanced prostate cancer patients and spouses: results from actor-partner interdependence models
Source: Support Care Cancer. 2022 May 13;30(8):6985–93. doi: 10.1007/s00520-022-07100-8 (PMC9213378; doi:10.1007/s00520-022-07100-8)
Supplement: Supplementary file 3 — (DOCX 15.7 kb) [file 520_2022_7100_MOESM3_ESM.docx]

|  | estimated parameters | Chi² (df), *p* | CFI | RMSEA | SRMR |
| --- | --- | --- | --- | --- | --- |
| **GAD** | | | | | |
| model 1a | 14 | - | 1.000 | .000 | .000 |
| model 2a | 14 (2 fixed) | 7.44 (2), .02* | 0.937 | .168 | .073 |
| model 3a | 14 | - | 1.000 | .000 | .000 |
| **FoP** | | | | | |
| model 1b | 14 | - | 1.000 | .000 | .000 |
| model 2b | 14 (2 fixed) | 4.53 (2), *.10** | 0.977 | .106 | .050 |
| model 3b | 14 | - | 1.000 | .000 | .000 |
| **Depression** | | | | | |
| model 1c | 14 | - | 1.000 | .000 | .000 |
| model 2c | 14 (2 fixed) | 2.04 (2), *.36* | 1.000 | .015 | .043 |
| model 3c | 14 (6 fixed) | 5.32 (6), *.50* | 1.000 | .000 | .093 |

Supplement 3: Goodness of fit parameters

*Note:* Values of each model 1-3 were corrected with the Yuan-Bentler-correction for non-normally distributed data, values of models 4 were bootstrapped.

*Recommended alpha level = .20 (see ^1^)

Reference

^1^ Kenny DA, Ledermann T. Detecting, measuring, and testing dyadic patterns in the actor-partner interdependence model. J Fam Psychol. 2010;24: 359-366.
