# Supplementary material for: Health-related quality of life of advanced prostate cancer patients and spouses: results from actor-partner interdependence models
Source: Support Care Cancer. 2022 May 13;30(8):6985–93. doi: 10.1007/s00520-022-07100-8 (PMC9213378; doi:10.1007/s00520-022-07100-8)
Supplement: Supplementary file 4 — (DOCX 16.5 kb) [file 520_2022_7100_MOESM4_ESM.docx]

Supplement 4: Correlation matrix

|  | *GAD patient* | *GAD spouse* | *FoP patient^1^* | *FoP spouse^1^* | *PHQ patient* | *PHQ spouse* | *HRQoL patient* | *HRQoL spouse* |
| --- | --- | --- | --- | --- | --- | --- | --- | --- |
| *GAD patient* | 1.00 | 0.24* | 0.62*** | 0.35*** | 0.74*** | 0.45*** | -0.53*** | -0.28** |
| *GAD spouse* | 0.24* | 1.00 | 0.35*** | 0.71*** | 0.37*** | 0.68*** | -0.47*** | -0.49*** |
| *FoP patient^1^* | 0.62** | 0.35*** | 1.00 | .57*** | 0.57*** | 0.48*** | -.43*** | -.29** |
| *FoP spouse^1^* | 0.35** | 0.71*** | .57*** | 1.00 | 0.45*** | 0.57*** | -.46*** | -.45*** |
| *PHQ patient* | 0.74*** | 0.37*** | 0.57*** | 0.45*** | 1.00 | 0.53*** | -0.67*** | -0.39*** |
| *PHQ spouse* | 0.44*** | 0.68*** | 0.48*** | 0.57*** | 0.53*** | 1.00 | -0.41*** | -0.56*** |
| *HRQoL patient* | -0.53*** | -0.47*** | -.43*** | -.46** | -0.67*** | -0.41*** | 1.00 | .49*** |
| *HRQoL spouse* | -0.28** | -0.49*** | -.29** | -.45*** | -0.39*** | -0.56*** | .49*** | 1.00 |

Note: GAD (anxiety scale) and PHQ (depression scale) of the Patient Health Questionnaire-4 (PHQ-4); FoP = fear of progression; HRQoL = health-related quality of life.

*** *p* ≤ .001, ***p* ≤.01; * *p* ≤ .05.; *N* = 96; ^1^ *N* = 95 (missing value).
